# Cap‐independent translation: A shared mechanism for lifespan extension by rapamycin, acarbose, and 17α‐estradiol

**DOI:** 10.1111/acel.13345

**Published:** 2021-03-20

**Authors:** Ziqian Shen, Abby Hinson, Richard A. Miller, Gonzalo G. Garcia

**Affiliations:** ^1^ Department of Molecular, Cellular, and Developmental Biology University of Michigan College of Literature, Science, and the Arts Ann Arbor Michigan USA; ^2^ Department of Pathology University of Michigan School of Medicine Ann Arbor Michigan USA; ^3^ University of Michigan Geriatrics Center Ann Arbor Michigan USA

**Keywords:** 17α‐estradiol, acarbose, aging, protein translation, rapamycin, signal transduction

## Abstract

We hypothesized that rapamycin (Rapa), acarbose (ACA), which both increase mouse lifespan, and 17α‐estradiol, which increases lifespan in males (17aE2) all share common intracellular signaling pathways with long‐lived Snell dwarf, PAPPA‐KO, and Ghr−/− mice. The long‐lived mutant mice exhibit reduction in mTORC1 activity, declines in cap‐dependent mRNA translation, and increases in cap‐independent translation (CIT). Here, we report that Rapa and ACA prevent age‐related declines in CIT target proteins in both sexes, while 17aE2 has the same effect only in males, suggesting increases in CIT. mTORC1 activity showed the reciprocal pattern, with age‐related increases blocked by Rapa, ACA, and 17aE2 (in males only). METTL3, required for addition of 6‐methyl‐adenosine to mRNA and thus a trigger for CIT, also showed an age‐dependent increase blunted by Rapa, ACA, and 17aE2 (in males). Diminution of mTORC1 activity and increases in CIT‐dependent proteins may represent a shared pathway for both long‐lived‐mutant mice and drug‐induced lifespan extension in mice.

## Graphical abstract

ACA, Rapamycin and 17alfa Estradiol downregulate mTORC1 activity and upregulation of the Cap‐Independent Translation leading to increases in the levels of cap‐Independent associated proteins (involved in mitochondrial function and stress resistance), suggesting a possible mechanism for lifespan extension.

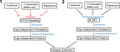

**Abbreviations**
4EBP1: eukaryotic translation initiation factor 4E binding protein 1
ACA: Acarbose
AKT: Protein Kinase B
Conex‐20: GAP junction protein 20
eIF4E: eukaryotic translation initiation factor 4E
Hsp70: heat shock proteins 70
METTL14: nuclear N6‐adenosine‐methyltransferase 14
METTL3: nuclear N6‐adenosine‐methyltransferase 3
MGMT: O‐6‐methylguanidine‐DNA methyltransferase
mTOR: mechanistic target of rapamycin
mTORC1: mechanistic target of rapamycin complex 1
mTORC2: mechanistic target of rapamycin complex 2
NDRG1: N‐myc downstream regulated gene‐1
p38: p38 mitogen activated protein kinase
Rapa: rapamycin
S6: ribosomal protein S6
TFAM: mitochondrial transcriptional factor A

## 1 INTRODUCTION

Several dietary and pharmacological treatments are known to extend lifespan, including rapamycin [Rapa, (Harrison et al., 2009; Miller et al., 2011, 2014)], acarbose [ACA, (Miller et al., 2014)], and 17‐α‐estradiol [17aE2, (Harrison et al., 2014; Strong et al., 2016)]. The mechanisms by which these treatments lead to lifespan extension are not well understood. Rapa inhibits the activity of the mammalian target of rapamycin (mTOR), leading at optimal doses to 20%–25% lifespan extension in male and female mice (Garratt et al., 2016; Miller et al., 2014; Zhang et al., 2014). ACA is an inhibitor of the α‐glucosidase hydrolase enzymes and α‐amylases, enzymes that digest carbohydrates in the small intestine, leading to reduction in glucose absorption and in peak glucose levels in blood (Madar & Hazan, 1993; Madar et al., 1994). ACA extends lifespan by around 20% in males and around 5% in female mice (Harrison et al., 2014). 17aE2 is a non‐feminizing steroid that has a reduced affinity for the classical estrogen receptors (Harrison et al., 2014). 17aE2 has reproducible and robust effects on male median and maximum lifespan, with no lifespan effect in females (Strong et al., 2016).

Studies of acute treatments of Rapa “in vivo” have focused mainly on transcriptional changes linked to reduction in mTORC1 activity (Kim & Guan, 2019). However, rapamycin also affects the rate and scope of protein translation (Roux & Topisirovic, 2018). Rapa can decrease cap‐dependent mRNA translation via inhibition of the phosphorylation of the ribosomal protein S6 [S6_(pS235)_] as well as phosphorylation of the eukaryotic translation initiation factor 4E‐binding protein 1 [4EBP1_(pT37)_, (Beretta et al., 1996; Thoreen et al., 2012)]. Declines in 4EBP1_(pT37)_ (or increases in 4EBP1 protein) levels have been shown to reduce cap‐dependent translation and, indirectly, increase the levels of cap‐independent translation or CIT (Lacerda et al., 2017). The function and relevance of CIT are not well understood, but there is evidence for CIT as a modulator of enhanced stress resistance, metabolic processes, and development [i.e., (Dennis et al., 2013; Lacerda et al., 2017)]. Many of the CIT mechanisms involve specific elongation and initiation factors in addition to proteins that recognize sequences or modifications in the sequence of 5′UTR, including 6‐methyl‐adenosine residues (“m6A”) of a select subset of mRNAs (Lacerda et al., 2017; Lence et al., 2017). In addition, inhibition of cap‐dependent translation, for example inhibiting mTOR with Rapa, can initiate translation of these m6A‐containing mRNA, leading to increases in protein independent of any changes in transcription of the corresponding mRNA. CIT target proteins upregulated “in vivo” and “in vitro” by Rapa treatments include mitochondria and stress proteins, such as O‐6‐methylguanidine‐DNA methyltransferase (MGMT), N‐myc downstream regulated gene‐1 (NDRG1), mitochondrial transcriptional factor A (TFAM), and heat shock protein 70 (Hsp70) (Dominick et al., 2017; Dominissini et al., 2012; Lacerda et al., 2017; Niu et al., 2013; Ozkurede et al., 2019). Other mechanisms that could upregulate protein levels, such as protein stability and cap‐dependent translation, do not seem to play a significant role for this set of proteins, when mRNA levels do not change. Our published results show, for example, that the rate of turnover (degradation) of MGMT, TFAM, NDRG1, LONP1, and YTHDF1 is actually higher in GHR‐KO and Snell dwarf fibroblasts than in control cells (e.g., Figure 2 in Ozkurede et al., 2019). Although the mechanism for higher turnover is not yet known, the results argue strongly against the idea that high levels of these proteins in cells of mutant mice reflect slower protein degradation (Dominick et al., 2015; Dominick et al., 2017; Smalley, Chalmers, & Morley, 2014); Ozkurede et al., 2019).

ACA affects post‐prandial glucose transients, and it is unclear how these in turn lead to health benefits in mice, but in liver there is evidence suggesting upregulation of protein B kinase (AKT) and n‐myc downstream regulated protein‐1 (NDRG1) phosphorylation levels, suggesting that upregulation of the mTORC2 pathway could be beneficial for glucose homeostasis (Garratt et al., 2017). In addition, ACA can regulate the extracellular signal‐regulated kinase (ERK) in adipose tissue (Perez et al., 2008).

17aE2 can also enhance glucose homeostasis in male mice and stimulate increases in hepatic mTORC2 activity, a key modulator of glucose homeostasis; but these effects are seen in male mice only (Garratt et al., 2017). Little is known about the molecular basis for sex‐specific effects of 17aE2 on glucose control, mTORC2, and lifespan. Because each of these three interventions [ACA, Rapa, and 17aE2 (in males only)] can extend mouse lifespan, we hypothesized that they might share common effects on cell biology in one or more tissues relevant to tissue health, metabolic homeostasis, and lifespan extension. A recent meta‐analysis of transcriptional changes induced by ACA, Rapa, and 17aE2 did not lead to a clear model of what common pathways might link these interventions to lifespan extension (Tyshkovskiy et al., 2019). In addition, it remains unknown whether chronic ACA, Rapa, or 17aE2 exposure at doses used for lifespan extension leads to modulation of mTOR downstream signaling, cap‐dependent translation, or CIT in mouse tissues. Therefore, we wished to test the idea that these three drugs might modulate mTORC1 signaling in ways that led to increases in CIT activity and thus to translation of select subsets of CIT‐eligible mRNAs. This idea was triggered by our observations that three varieties of slow‐aging mice with diminution of GH/IGF1 signaling, that is, Snell Dwarf, Ghr−/−, and PAPPA‐KO mice, all have a reduction in mTORC1 and enhancement in CIT activity (Dominick et al., 2017). Here, we evaluate ACA, Rapa, and 17aE2 effects on age‐related changes in key intracellular signaling pathways involved in translation, including mTOR, and effects of these drugs on the proteins regulated by CIT in two tissues: liver and kidney. Our new results suggest a common mechanism shared by these three interventions.

## 2 RESULTS

### 2.1 ACA, Rapa, and 17aE2 enhance CIT activity

**FIGURE 1:**
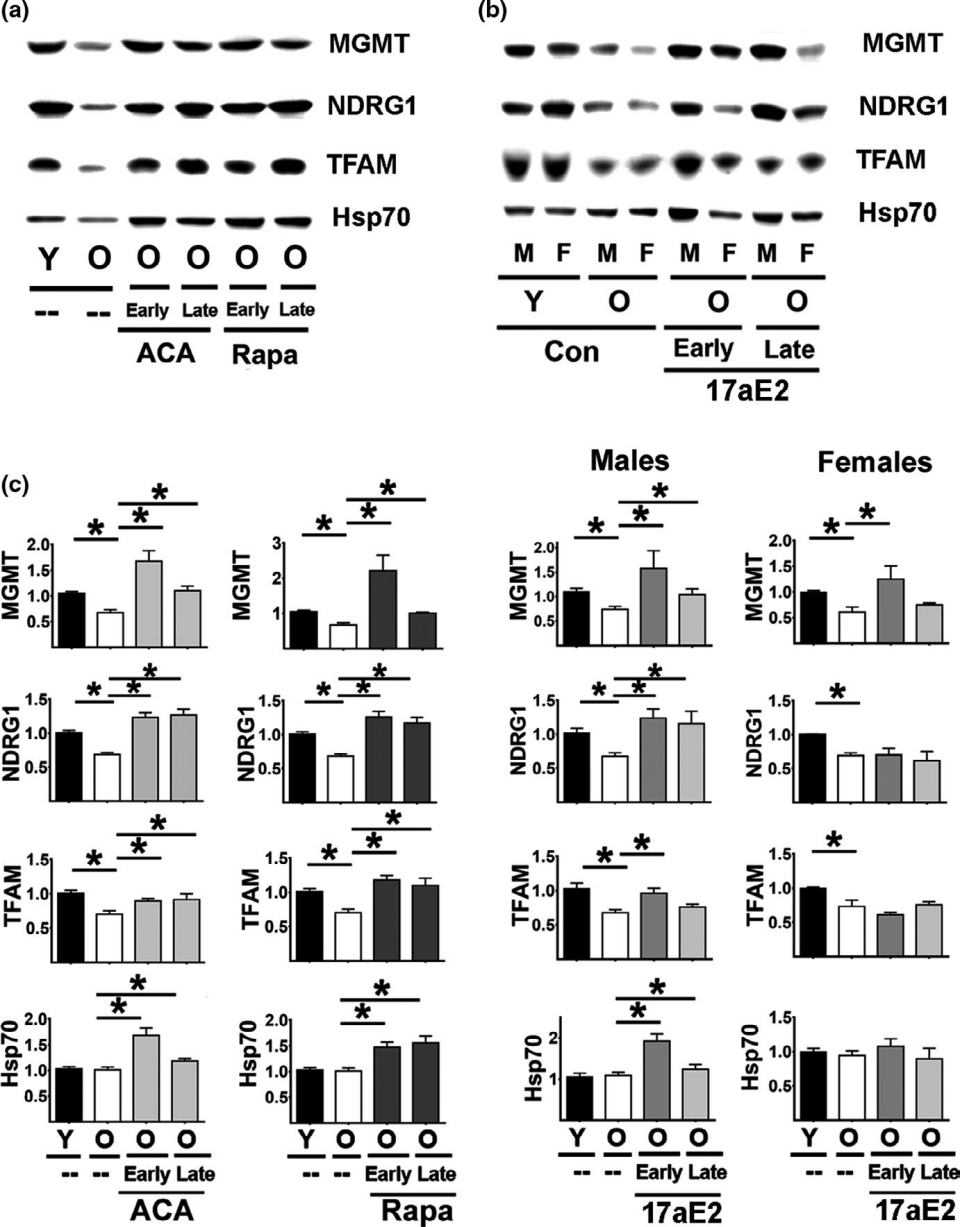
Effect of age, ACA, Rapa, and 17aE2 treatments on hepatic levels of several proteins regulated by CIT. (a) Representative Western blots for CIT‐regulated proteins: MGMT, NDRG1, TFAM, and Hsp70 in liver samples from young untreated (Y), old untreated (O), old treated with ACA or Rapa (O, early and late treatments). (b) Representative Western blots showing the effects of age and 17aE2 (early and late) treatments separated by sex (males = M, females = F) for the same CIT‐regulated proteins. (c) Bar graphs represent the mean ± SEM for each of the CIT‐regulated proteins in liver samples obtained from 16 young and 16 old mice plus at least 8 mice for each of the ACA and Rapa groups. Data for 17aE2 were obtained from 8 young and 8 old mice and a minimum of 4 for each male and female group. All values have been normalized to young control females as described in Methods section. The (*) indicates statistical significance (*p* < 0.05) in a *t* test between the indicated pair of groups

We collected livers and kidneys of young mice (6 months of age, Y), old mice (22 month of age, O), and mice treated with either ACA, Rapa, or 17aE2 from 6 to 22 months of age (O, Early) or from 18 to 22 months of age (O, Late). (See Figure S1 for a visualization of the experimental design). Cell lyses were obtained, and actin levels estimated by Western blot. Then, each sample was adjusted to identical levels of actin by serial dilution to normalize for batch and extraction differences [for details of batch adjustment and normalization see methods section as previously described (Ozkurede et al., 2019)]. Then, CIT activity was evaluated by measuring the levels of CIT target proteins MGMT, NDRG1, TFAM, Hsp70, and Connexin20 by Western blots (see Figure 1a). Values for each CIT target protein were normalized to the results for the young female control in the same Western blots to determine relative fold changes as previously described (Dominick et al., 2017). Lastly, a comparative statistical analysis of each CIT target was performed by a two‐way ANOVA to determine effect of age, sex, and interactions. The (age x sex) interaction for Rapa or ACA treatment was not statistically significant, and therefore, males and females were combined for each treatment. In contrast, a statistically significant interaction term was seen for most groups exposed to 17aE2; see Table S1 for details. For this reason, results for males and females are analyzed and reported separately for 17aE2‐treated mice.

CIT proteins MGMT, NDRG1, TFAM, and Hsp70 were evaluated in liver and kidney samples. In addition, kidney samples were tested for levels of Connexin20, which is not detectable in liver. We noted a significant age‐related decline in MGMT, NDRG1, and TFAM in liver, but no decline in Hsp70 (Figure 1 panel c). Treatments with ACA (starting early, i.e., at 6 months, or late, i.e., at 16 months) or Rapa (early or late) can significantly increase MGMT, NDRG1, and TFAM to levels equal to or higher than levels seen in young mice. In addition, ACA and Rapa (starting at either age) can also significantly increase Hsp70 above the levels seen in old control mice. In males, 17aE2 also increases all four of these CIT proteins, similar to the effects of ACA and Rapa. In contrast, females show no effects of 17aE2 on NDRG1, TFAM, or Hsp70. MGMT was upregulated in 17aE2‐treated females, but only if mice were exposed from an early age (Figure 1c). The sex‐specific effects of 17aE2 are in accord with similar sexual dimorphism for lifespan and a range of age‐sensitive physiological endpoints (Garratt et al., 2017; Harrison et al., 2014).

**FIGURE 2:**
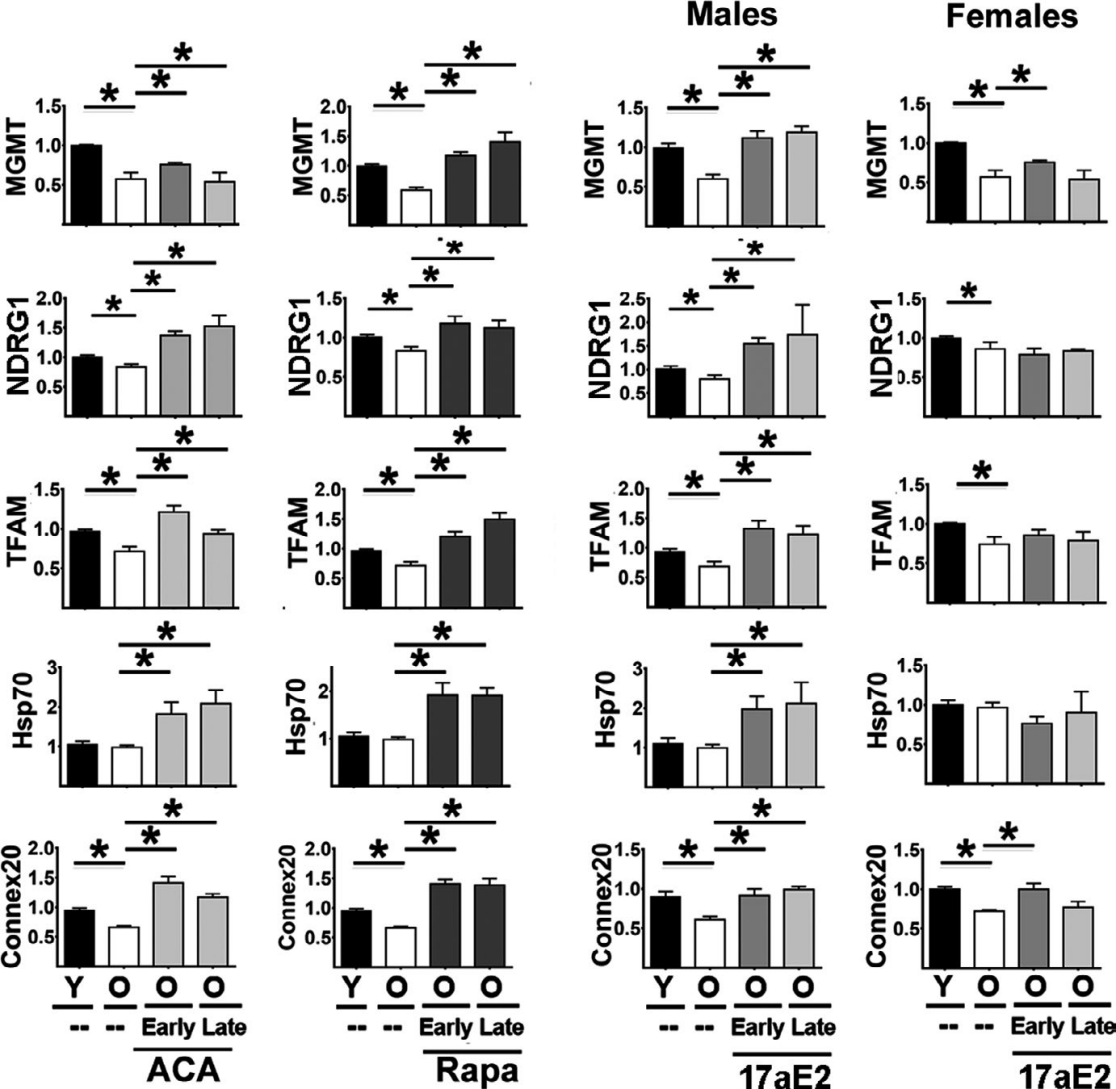
Effect of age, ACA, Rapa, and 17aE2 treatments on kidney levels of CIT‐regulated proteins. Bar graphs represent the mean ± SEM for each of the CIT targets plus connexin20 in kidney samples from the same numbers of mice described in Figure 1. All values have been normalized to young control females as described in Methods section. The (*) indicates statistical significance (*p* < 0.05) in a *t* test between the indicated pairs of groups

Analysis in kidneys showed a similar pattern (Figure 2 and Table S1), with age‐related declines in the expression of CIT targets MGMT, NDRG1,and TFAM but not in Hsp70. ACA and Rapa, started early or late, significantly increased levels of MGMT, NDRG1, TFAM, and Hsp70 with respect to old controls, again in good agreement with the results in liver. 17aE2‐treated males show higher levels of all four of these proteins. In contrast, females show no effects of 17aE2 on NDRG1, TFAM, or Hsp70. 17aE2 increases MGMT levels in females only when started at an early age, and the effect on MGMT, though significant, is small. Expression of Gap Junction 20 (connexin‐20) is another important marker of CIT activity (Zeitz et al., 2019). Connexin‐20 is produced exclusively by alternative translation from full length Gap Junction 46 mRNA (connexin‐46) by a CIT mechanism (Zeitz et al., 2019). Higher levels of connexin‐20 thus do not depend on connexin‐46 mRNA levels but rather on higher CIT activity (Zeitz et al., 2019). Aging leads to a significant decline in connexin‐20 in both sexes (Figure 2 bottom panels). ACA and Rapa significantly enhance expression of connexin20 as they do for the other CIT targets. 17aE2 leads to significantly higher conexin‐20 in males but does so in females only if started at an early age.

**FIGURE 3:**
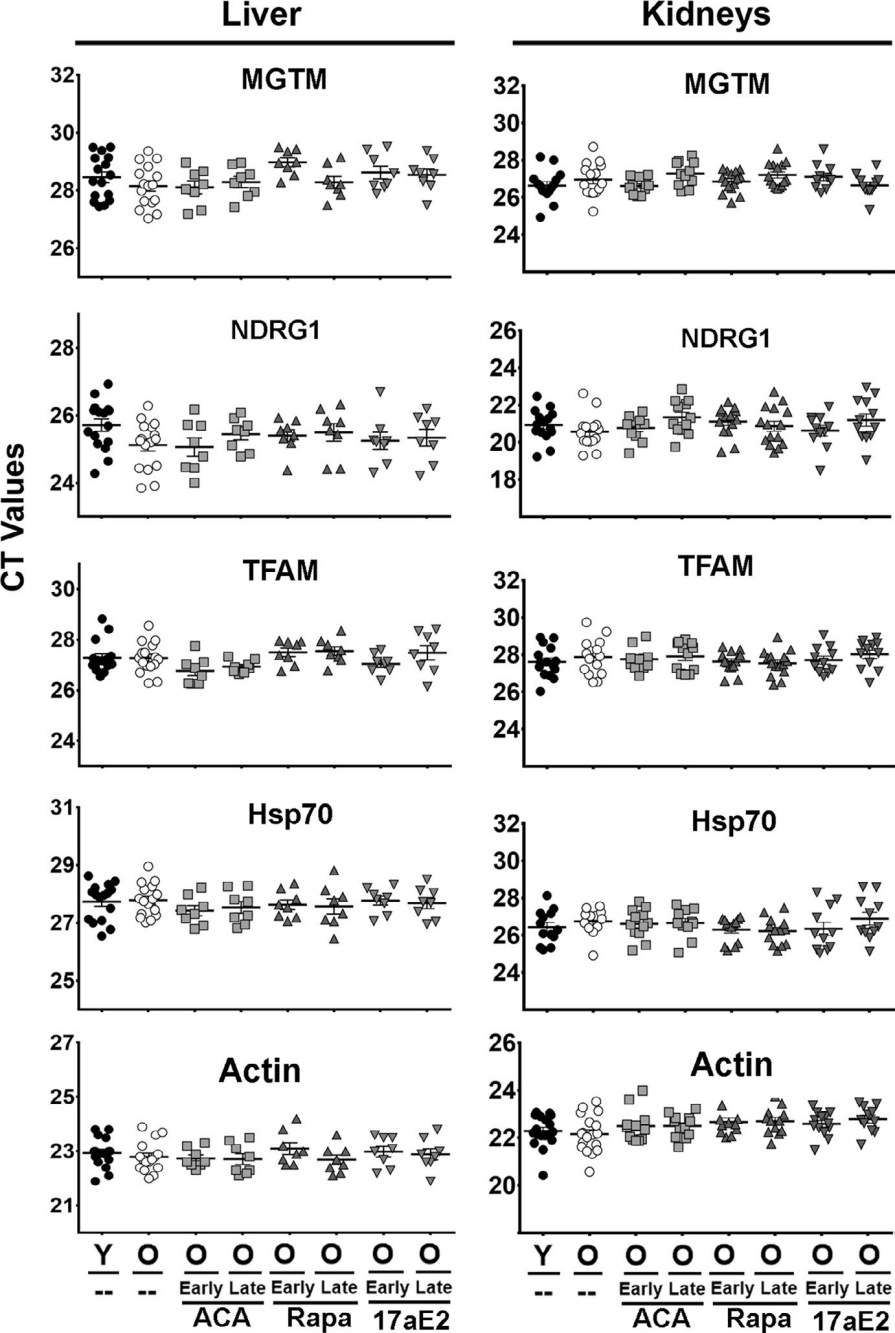
No significant differences in the mRNA levels of CIT targets associated with age or drug treatments in liver and kidney. The scatter plot shows a comparison of CT values and mean ± SEM (horizontal bar) of each mRNA between the groups from 16 young and 16 old mice plus a minimum of 8 mice treated with ACA, Rapa, and 17aE2 (earl or late) per group. Each symbol represents a different mouse

To test whether any of these changes are the results of transcriptional upregulation, we extracted mRNA from the same mice and performed qRT‐PCR analysis of the mRNA levels for MGMT, NDRG1, TFAM, and Hsp70 in liver and kidney. The levels of connexin‐20 mRNA were not quantified due to high homology with multiple members of the connexin‐46 family. Analysis by two‐way ANOVA suggested no significant differences in their mRNA levels by effect of age, sex, or time of treatments (early or late); see Figure 3. The result suggests that the effects of age and drug treatment of these proteins reflect post‐transcriptional mechanisms, such as enhanced CIT activity.

### 2.2 Effects of ACA, Rapa, and 17aE2 on mTORC1 signaling

**FIGURE 4:**
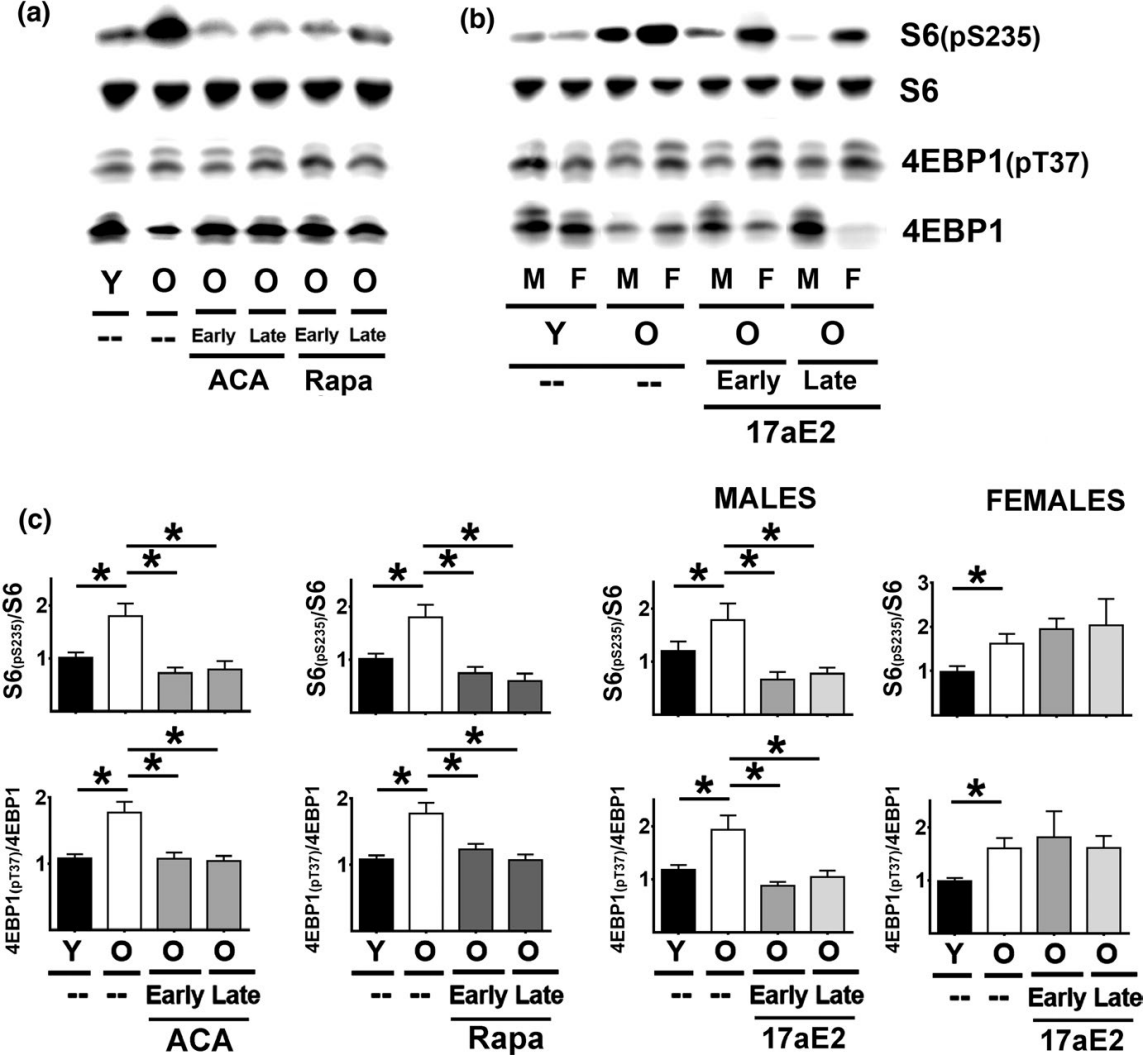
Effect of age and ACA, Rapa, and 17aE2 treatments on hepatic mTORC1 activity. (a) Representative Western blots of S6, S6_(pS235)_, 4EBP1_(pT37),_ and 4EBP1 levels in livers for each age and drug treatment as described in Figure 1. (b) Representative Western blots of the same mTORC1 substrates and the effects of age and 17aE2 (early and late) treatments, shown separately for each sex (males = M, females = F) (c) Bars represent the mean ± SEM of each S6 _(pS235)_/S6 and 4EBP1_(pT37)_/4EBP1 ratios obtained from the same numbers of mice described in Figure 1c. All values have been normalized to young control females as described in the Methods section. The (*) indicates statistical significance (*p* < 0.05) in a *t* test between the groups indicated by the bar

mTORC1 activity was assessed by measuring the level and phosphorylation status of two mTORC1 substrates: S6 protein and its phosphorylation sites at serine 235/236 [S6_(pS235)_], and 4EBP1 and its phosphorylation sites at threonine 37/46 [4EBP1_(pT37)_] by immunoblotting (Figure 4 panels A and B). Two‐factor ANOVA revealed significant sex‐by‐drug interactions only for 17aE2, and for this reason, evaluation of Rapa and ACA effects involved data pooled across sex (Table S2), similar to the analyses used above for CIT target proteins.

In liver samples, there were no significant effects of age, ACA, Rapa, or 17aE2 treatments on the levels of S6 protein. In contrast, we noted a significant age‐related increase in the phosphorylated form of S6_(pS235)_ for both sexes (Figure S2A). ACA and Rapa (both early and late) significantly reduced S6_(pS235)_ independently of sex; 17aE2 also reduced S6_(pS235)_ but only in males (Figure S2A). Because the ratio of S6_(pS235)_/S6 provides an index of mTORC1 activity, we examined this ratio as a function of age and treatments. The results (Figure 4b) show an age‐dependent increase in the S6_(pS235)_/S6 ratio and, by implication, mTORC1 activity. ACA and Rapa (both early and late) reduce this ratio, and 17aE2 does so in male mice only (Figure 4c).

The 4EBP1_(pT37)_/4EBP1 ratio showed the same pattern of age, sex, and drug effects: age‐related increases in both sexes, reversed or blunted by Rapa and ACA in both sexes and by 17aE2 in males only, regardless of the age at which drug exposure was initiated. In contrast to the phosphorylation ratio for S6, the age and drug effects for the 4EBP1_(pT37)_/4EBP1 ratio do not reflect changes in the phosphorylation level of 4EBP1_(pT37)_ per se, but instead reflect changes in the level of total 4EBP1 protein (see Figure S2B). It is possible that the 18 h. of fasting imposed on mice prior to euthanasia may have led to reduction in the 4EBP1_(pT37)_ levels as noted previously by us and others in several mouse models (Dominick et al., 2015, 2017). In addition, there was a significant age‐related reduction in 4EBP1 protein levels (Figure S4B) and also that ACA and Rapa (both early and late) significantly enhanced the levels of 4EBP1 protein, perhaps by blocking or reversing the age‐related decline. 17aE2 (early and late) can also enhance the levels of 4EBP1, but only in males (Figure S2B).

**FIGURE 5:**
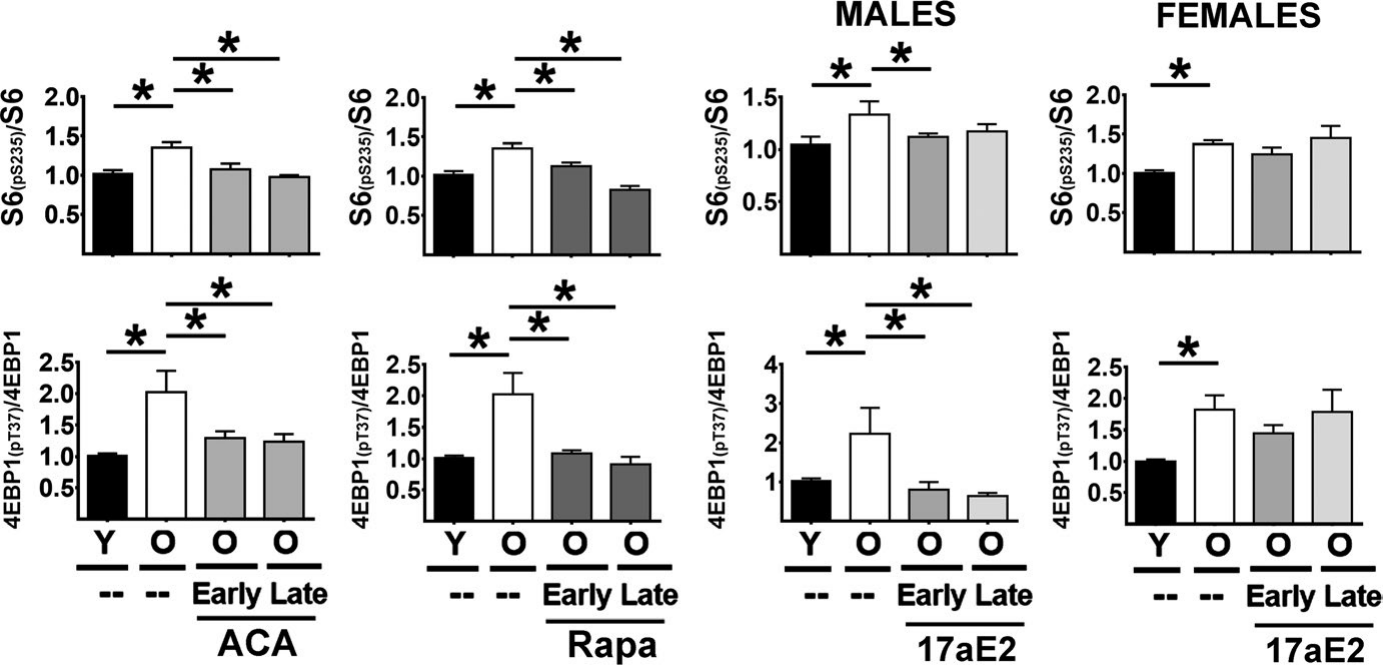
Effect of age and ACA, Rapa, and 17aE2 treatments on mTORC1 activity in kidneys. Bar graphs represent the mean ± SEM of each S6_(pS235)_/S6 and 4EBP1_(pT37)_/4EBP1 ratios obtained from the same numbers of mice described in Figure 1. All values have been normalized to young control females as described in Methods section. The (*) indicates statistical significance (*p* < 0.05) in a *t* test between the indicated group

The kidneys showed an identical pattern, with significant age‐related increases in both S6_(pS235)_/S6 and 4EBP1_(pT37)_/4EBP1 ratios, suggesting increases in mTORC1 activity (Figure 5), with ACA and Rapa leading to significant reduction in both ratios in both sexes, regardless of the age at which treatment was started. 17aE2 led to a similar reduction in both ratios, but in male mice only, as seen in the liver data sets. Thus, these drugs appear to diminish mTORC1 activity in kidney as in liver, and for 17aE2 in a sex‐specific fashion. It is important to note, however, that in kidney, as in liver, we see no significant effects in the amount of 4EBP1_(pT37)_ from age or drug treatments (Figure S2D). The main effects under our test conditions reflect age‐related declines in 4EBP1 and its reversal or prevention by ACA and Rapa as well as by 17aE2 in males only (Figure S2D).

To test whether the effects on 4EBP1 protein levels were the result of transcriptional changes, we used qRT‐PCR to measure the relative levels of mRNA in the same samples used for the immunoblotting analyses. Neither S6 nor 4EBP1, in liver or kidney, showed alteration in the mRNA levels related to age, sex, or drug treatments (Figure S3) suggesting that alteration in 4EBP1 protein levels reflect post‐transcriptional mechanisms, potentially involving elevation of CIT or alterations in the translation machinery leading to enhanced 4EBP1 levels. The enhanced CIT activity with a lack of changes in the corresponding mRNAs is similar to what have been reported in several slow‐aging mice models (Ozkurede et al., 2019).

### 2.3 Effects of ACA, Rapa, and 17aE2 on METTL3 and METTL14 levels

**FIGURE 6:**
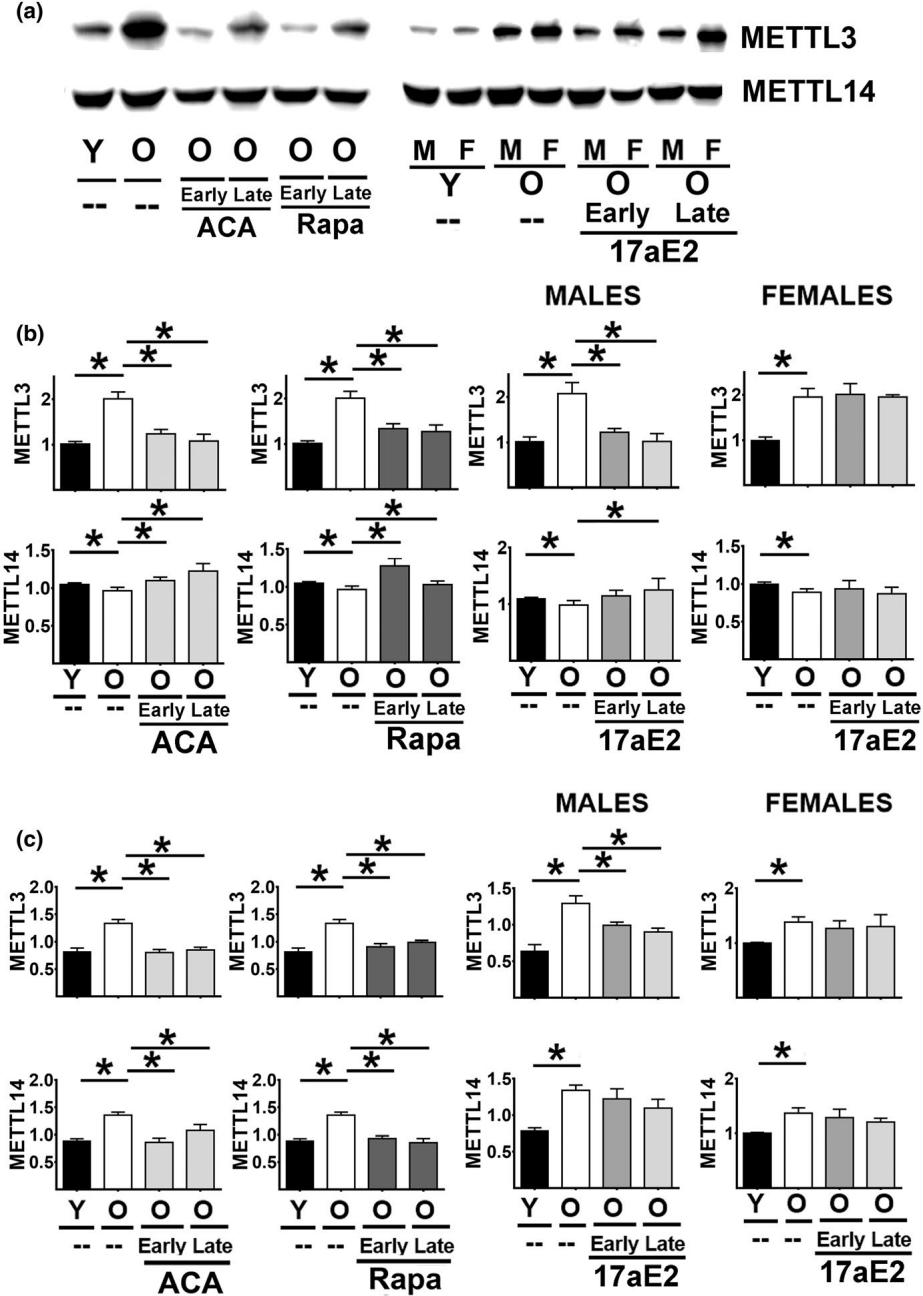
Effect of age and ACA, Rapa, and 17aE2 on METTL3 and METTL14 levels in liver and kidney. (a) Representative Western blots of METTL3 and METTL14 in livers from same numbers of mice described in Figure 1. (b) Mean ± SEM of METTL3 and METTL14 in livers as described in Figure 1c. (c) Mean ± SEM of METTL3 and METTL14 in kidney as described in Figure 1c. All values have been normalized to young control females as described in the Methods section. The (*) indicates statistical significance (*p* < 0.05) in a *t* test between the indicated pairs of groups

METTL3 and METTL14 are the key enzymes responsible for adding the m6A modification to mRNA that regulates CIT (Ozkurede et al., 2019; Wu et al., 2016). Several slow‐aging mice models, including Snell Dwarf, Ghr−/−, mice and PAPPA mice, have shown tissue specific increases in the levels of METTL3 and METTL14, suggesting upregulation of the m6A pathway and CIT (Ozkurede et al., 2019). To see whether similar upregulation of the m6A pathway takes place by the ACA, Rapa, or 17aE2, liver and kidney samples from the same cohort used for Figures 1 and 2 were analyzed for levels of METTL3 and METTL14 (Figure 6 panel a). The two‐way ANOVA (Table S3) suggested no significant effect of sex in the changes induced by age, ACA, and Rapa treatments; therefore, males and females were pooled for further analysis (Table S3). Again, 17aE2 showed a significant sex interaction effect, and males and females were therefore analyzed separately (Table S3).

The liver samples (Figure 6 panel a) showed a significant age‐related increase in METTL3 levels, and a small but significant downregulation of METTL14. The aging effect on METTL3 was blocked, or perhaps reversed, by Rapa and ACA, and by 17aE2 in male mice, regardless of the age at which drug treatment was started. The small age‐related decline in METTL14 was also reversed by Rapa and ACA. Late‐start 17aE2 showed the same effect in male mice, but not in females. METTL3 levels in kidney showed the same pattern as seen in liver, that is, age‐related increases in both sexes, opposed by Rapa and ACA, and by 17aE2 in males only (Figure 6 panel c). Aging increases METTL14 in kidney, in both sexes, an effect opposite in direction to the METTL14 age effect in liver, and this increase is mitigated by Rapa and ACA, regardless of the age at which drug was started. 17aE2 has no effect on kidney METTL14 in either sex. The mRNA data (Figure S4) found no effects of age, sex, or drug treatment on METTL3 or METTL14 mRNA, implying that the changes in protein level documented in Figure 6 reflect post‐transcriptional mechanisms, possibly involving differential mRNA translation.

## 3 DISCUSSION

Several genetic models of slow aging, such as Snell dwarf, Ghr−/−, and Pappa‐KO mice, show increases in levels of proteins regulated by CIT, including those involved in mitochondrial function (i.e., TFAM) and cellular stress resistance, such as MGMT and NDRG. The CIT‐dependent increases in translation of these mRNAs might be caused by one or several factors in combination, potentially including a decline in cap‐dependent translation and/or increases in the pathways involved in adding, reading, or removal of m6A residues in the 5′UTR of the corresponding mRNA (Ozkurede et al., 2019). Snell Dwarf, Ghr−/−, and PAPPA‐KO mice also show a significant reduction in mTORC1 signaling that could lead to declines in cap‐dependent translation and upregulation of relative or absolute levels of CIT (Dominick et al., 2017; Ozkurede et al., 2019). Short‐term (24–48 h) exposure of cells in culture or young adult mice to Rapa also augments levels of CIT‐dependent proteins, presumably by acute inhibition of mTORC1 and mTORC1‐dependent cap‐dependent translation (Beretta et al., 1996; Li et al., 2002; Sun et al., 2011). Because Rapa extends healthy lifespan of mice when given in food from early adult life or starting as late as 20 months, these data suggest that higher CIT may be one possible mechanism for anti‐aging effects shared by genetic and at least some drug‐dependent interventions. The current study tests this speculation by evaluation of CIT target proteins in mice treated chronically with Rapa, and in mice given either ACA or 17aE2, each of which leads to dramatic lifespan increases in males with either partial or no lifespan effects in female mice (ACA and 17aE2, respectively) (Garratt et al., 2017; Harrison et al., 2009). Data on this point could test the central idea of CIT as a shared mechanism and begin to provide molecular details as to the mechanism of action of Rapa, ACA and 17aE2. Our data in Figures 1 and 2 support the idea of CIT as a shared anti‐aging mechanism, showing enhanced CIT activity and upregulation of CIT target proteins including MGMT, NDRG1, TFAM, Hsp70, and Connexin20 in kidneys. It is also possible that Rapa, ACA, and 17aE2 could alter cell behavior by acting through pathways that do not involve mTORC1 signaling. ACA, for example, modifies daily peak glucose levels and insulin sensitivity and could alter liver and kidney responses through effects on adipokines, brain‐derived neuropeptides, or other mediators. The cellular target of 17aE2 is unknown but could in principle involve direct or indirect changes in steroid‐dependent inter‐ and intracellular activities. Independently of these possible alterations in signaling pathways, we noted age‐related declines in each of these proteins, except Hsp70, in liver and kidney tissues. It is not clear whether the drug effects represent delay in these age‐dependent changes, or, alternatively, reversal soon after drug treatment begins. The similarity in outcomes in mice given Rapa, ACA, or 17aE2 (for male mice) starting at 6 months or at 18 months suggests that effects may at least partly reflect reversal of age‐related change, rather than deceleration of a life‐long trend, but more information as to the onset and durability of drug effect is needed to clarify this issue. The Hsp70 data suggest that Rapa, ACA, and 17aE2 effects are not restricted to those proteins that show age‐related change, and thus demonstrate that drug treatments can augment CIT target proteins that are age‐insensitive. It is of interest that 17aE2, started at the earlier age, has partial effects even in females, with upregulation of MGMT (liver and kidneys) and connexin20 levels (kidneys), though other targets such as NDRG1, TFAM, or Hsp70 are not affected in females even when 17aE2 is started early. Effects of 17aE2 in males on age‐sensitive traits, such as muscle weight and rotarod performance (Garratt et al., 2017; Harrison et al., 2019), are also fully induced when the drug is started as late as 16 months of age. The parallel results seen in liver and kidney suggest that each of the agents may have broad effects on multiple tissues, rather than effects on liver‐specific traits alone, although data on other tissues will help to test and refine this conclusion.

The mechanism of maintaining or increasing CIT by these treatments is not clear, but we hypothesize that declines in mTORC1 and enhanced CIT via reduction in the cap‐dependent translation could be one of the common mechanisms involved in blocking the age‐related effects on translation. Age increases the ratio of S6_(pS235)_/S6 and the ratio of 4EBP1_(pT37)_/4EBP1 (Figures 4 and 5) suggesting age‐related upregulation of mTORC1 activity, which could in turn lead to the declines in CIT noted in Figures 1 and 2. Though controversial, the increase in mTORC1 with age has been also reported by others in several mouse models [for a review, see Table 1 in Baar et al., 2016]. These age effects on mTORC1 function can vary depending on tissue, strain, and substrate tested. In fasted C57BL/6NNia mice, for example, one group (Baar et al., 2016) noted a small decline in mTORC1 phosphorylation of hepatic S6 between 6 and 24 months of age in males, but not in females. In contrast, using 4E‐BP1 as an mTORC1 substrate, this group found no change in males, but an increase between 6 and 24 months of age in females. Quite different patterns of age effect were seen in skeletal muscle and fat of this inbred stock by the same research team. Similarly, Houtkooper et al. (2011) showed data suggesting an age‐dependent decline in mTORC1 function in liver and muscle. Sorting out these discrepancies will require greater attention to design variables, including effects of (a) fasting, (b) timing of euthanasia with respect to most recent food intake, (c) age and health of the "old" mice tested, (d) differences between prepubertal mice, rapidly growing adolescent mice, and weight‐stable young adults, (e) evaluation of multiple mTORC1 substrates, and in particular (f) genetic background of the tested mice. Data presented in this paper were produced using UM‐HET3 mice, a genetically heterogeneous stock chosen to minimize the chances that findings would be idiosyncratic to specific inbred (and thus uniform and homozygous) backgrounds. We note, too, that the hypothesis that diminished mTORC1 activity can have beneficial effects on age‐related health changes may prove true whether age itself produces an increase, decrease, or no change in mTORC1 function.

Independent of this controversy, our data show that ACA, RAPA, and (in males) 17aE2 can significantly block or reverse these mTORC1 changes, suggesting that inhibition of mTORC1 and cap‐dependent translation may contribute to their ability to maintain or restore the high levels of CIT seen in young mice. It is notable in Figures 1 and 2 that for some of the CIT proteins, the levels in drug‐treated aged mice are above those seen in young controls. For Rapa, inhibition of mTORC1 activity may be a direct effect of Rapa on the multi‐enzyme complex, although more complex feedback pathways may become operational during 6–16 months of continuous Rapa treatment. For ACA, effects on mTORC1 activity may involve alterations in insulin production and sensitivity caused by blunting of post‐prandial glucose transients (Harrison et al., 2019; Madar et al., 1994, 1997; Perez et al., 2008), but this idea is speculative and at present not supported by direct experimental evidence. Pathways by which 17aE2 alters mTORC1 function are uncertain, though it is noteworthy that many of the effects of 17aE2 on male mice are absent when this agent is given to males that had been castrated at 3 months of age (Garratt et al., 2017), implying that testosterone or some related hormone is required for effective 17aE2 action.

We interpret the age‐ and drug‐related changes in phosphorylation ratio for S6 and 4EBP1 as evidence for effects on mTORC1 function, but we note that the S6 ratio reflects alterations in S6_(pS235)_ while the 4EBP1 ratio reflects alterations, instead, in the total amount of 4EBP1 protein, rather than its phosphorylated form 4EBP1_(pT37)_. We suspect that the use of mice fasted for 18 h. prior to euthanasia diminishes the phosphorylation level of 4EBP1 levels (Dominick et al., 2015, 2017), making it more difficult to assess effects of genotype or drug treatments. It is also noteworthy that ACA, Rapa, and 17aE2 (in males) can maintain or reverse age‐related declines in 4EBP1 protein (Figure S2). 4EBP1 is a main regulator of cap‐dependent translation, and its upregulation can enhance lifespan in lower organisms (Zid et al., 2009). Elevated 4EBP1 can have major beneficial effects in insulin signaling (Tsai et al., 2015). Therefore, tissue specific increases in 4EBP1 may lead to improved health and lifespan through multiple mechanisms, including a shift from cap‐dependent translation to CIT. Evaluation of other substrates for mTORC1 is likely to be informative, to learn more about effects of age, drug exposure, and sex on mTORC1 enzymatic activity, structural variation, and substrate selectivity. In addition, the data shown in this paper are all derived from two tissues, liver and kidney, and it will be important to see whether other tissues, such as adipose tissue, muscle, and various brain components, show a similar pattern or not.

Several slow‐aging mouse models, at 6 months of age, show age‐specific upregulation of the m6A pathway responsible for the methylation in position 6 of specific adenosine residues in mRNAs and upregulation of CIT (Ozkurede et al., 2019; Zhou et al., 2015). These changes include upregulation of METTL3 in liver and kidneys of Snell dwarf and Ghr−/− mice and upregulation of METTL14 in liver but not in kidneys. The data in Figure 5 show that age can significantly increase the levels of METTL3 protein in liver and kidney, while ACA, Rapa, and 17aE2 (males only) reduce the level of METTL3. The specific mechanism is unknown, and it is possible that these different drugs modify METTL3 through different pathways. Rapa‐mediated reduction in mTORC1 activity is the most obvious plausible hypothesis, and it is tempting to seek connections between ACA‐mediated blunting of glucose spikes and alterations in mTORC1 function. It is also possible that these agents could moderate or reduce inflammatory and stress‐related processes thought to increase with aging in some contexts (Mau et al., 2020; Rudovich et al., 2011; Sadagurski et al., 2017). Independent of how these treatments regulate the changes in METTL3 levels, the specific role of METTL3 in regulation of CIT remains controversial, with some data suggesting that higher METTL3 levels have detrimental effects by promoting translation of oncoproteins (Choe et al., 2018; Lin et al., 2016), while other data suggest a beneficial effect by increasing stress resistance (Lin et al., 2020; Wang et al., 2019). The controversy may be, in part, the result of tissue specific effects of METTL3 and the levels of other modulators of m6A pathways, including m6A removing enzymes like FTO and ALKBH5, and m6A‐reading factors like YTHDF1 and YTHDF2 (Wu et al., 2016). More data on these m6A‐related proteins will help us to understand the dynamics of m6A and the regulation of CIT by age, sexual dimorphism, and drug exposure. The case of METTL14 is more complex, with significantly upregulation by age in kidney and minor effects in liver. It is unknown what upstream signaling pathways regulate METTL3 and METTL14 levels, but in the slow‐aging Snell Dwarf and Ghr−/− mice the increases in both enzymes are associated with increases in their mRNA levels. These results suggest that the age‐related increases in both enzymes are the result of enhanced cap‐dependent translation, while drug‐induced declines in the UM‐HET3 mice shown in Figures 5 and 6 may be due to reduction in cap‐dependent translation where CIT may not be involved. In addition, the lack of alteration in METTL3 and METTL14 mRNA by age or drug treatment (Figure S4) seems to support this model. Nevertheless, the upregulation of CIT and its targets (MGMT, NDRG1, TFAM, and connexin20) does not seem to be dependent on the levels METTL3 and METLL14, regardless of the implications of the findings seen in the genetic models (Lence et al., 2017; Niu et al., 2013; Zhou et al., 2015).

## 4 MATERIALS AND METHODS

### 4.1 Mice and diets

Genetically heterogeneous UM‐HET3 mice were produced by a four‐way cross between CByB6F1 mothers and C3D2F1 fathers and housed as previously described (Garratt et al., 2017; Miller et al., 2014). Mice in breeding cages received Purina 5008 mouse chow, and weaned animals were fed Purina 5LG6. At 6 months of age, animals in different sibling groups were randomly allocated to control, ACA, Rapa, or 17aE2 treatments. All diets were prepared by TestDiet, Inc., a division of Purina Mills (Richmond, IN, USA), which also produces drug/food mixtures for the NIA Interventions Testing Program. Animals in the control group remained on the 5LG6 diet. Rapamycin was given as encapsulated Rapa (L.C. Laboratories, Woburn, MA) at a dose of 14 milligrams per kilogram of 5LG6 diet (14 ppm) as previously described (Harrison et al., 2009; Miller et al., 2011, 2014). ACA was purchased from Spectrum Chemical Mfg. Corp. (Gardena, CA) and used at a concentration of 1000 mg of ACA/kg of diet (1000 ppm) as previously described (Harrison et al., 2019). 17aE2 was purchased from Steraloids Inc. (Newport, RI, USA) and used at a dose of 14.4 mg per kilogram of 5LG6 diet (14.4 ppm) as previously described (Harrison et al., 2019). All these methods followed those recommended by the NIA Interventions Testing Program.

### 4.2 **Tissues harvested,** W**estern blots and qRT‐PCR analysis**

Tissues were harvested at the age indicated in Figure S1; tissues were harvested during the morning after 18 h of fasting. Tissues were frozen with liquid nitrogen and stored at −80°C. Cell lysates were prepared, after which equal amounts of protein were loaded for Western blot analysis of actin. Then, samples from each mouse were normalized to the same actin concentration present in young females control (untreated) mice and the specific proteins or phospho‐sites measured by Western blots as described previously (Ozkurede et al., 2019). Each Western Blot contained at least one sample for each group (young, old, ACA, Rapa or 17aE2 treatments for males and females). Data on band intensity were normalized with respect to young female controls for each blot, to allow combination of results from multiple Western blots. The specific antibodies used in the analysis are described in Table S1. qRT‐PCR from the same set of samples was performed as previously described (Ozkurede et al., 2019). qRT‐PCR probe sequences are provided in Table S2.

### 4.3 Statistics

All statistics were carried out in GraphPad Prism version 7.0. Data from animals treated with ACA, Rapa, and 17aE2 were analyzed separately, but the same young and aged control animals were used in each analysis. To normalize between different Western blots, we use young females as a reference as we previously described (Ozkurede et al., 2019). After normalization, the full data for each measured parameter were analyzed by two‐factor ANOVA, using a general linear model and a full factorial model, which included an effect of treatment (comparing control to either ACA or Rapa), an effect of sex (male or female), and an interaction between sex and treatment. Because for ACA and Rapa we found no significant interaction between treatments and sex, we pooled the data for males and females for each group to perform statistical analysis. For 17aE2, however, most endpoints had a significant Sex × Drug interaction term, and therefore males and females were analyzed separately. In the same way, the absence of a (Sex × Age) interaction term for comparisons of Young and Aged controls allowed us to pool across sex for tests of age effects on each endpoint. Each analysis of drug or sex effect then began with a one‐way ANOVA followed by *t* tests to evaluate specific contrasts of interest, that is, Young vs Old controls, and Old controls vs each drug‐treated group. Significance was evaluated using a criterion of *p* = 0.05 without adjustment for multiple comparisons.

## CONFLICT OF INTEREST

The authors have declared that no competing interests exist.

## AUTHOR CONTRIBUTIONS

Ziqian Shen, Abby Hinson, and Gonzalo G. Garcia carried out the experiments and data analysis with the help of Richard A. Miller. Richard A. Miller and Gonzalo G. Garcia wrote the manuscript, while Richard A Miller helped to supervise the project.

## Supporting information

Fig S1

Fig S2

Fig S3

Fig S4

Table S1‐S5

## Data Availability

All data that support the findings of this study are available from the corresponding author upon request.
