# Supplementary figures and images for: Cap‐independent translation: A shared mechanism for lifespan extension by rapamycin, acarbose, and 17α‐estradiol
Source: Aging Cell. 2021 Mar 20;20(5):e13345. doi: 10.1111/acel.13345 (PMC8135077; doi:10.1111/acel.13345)

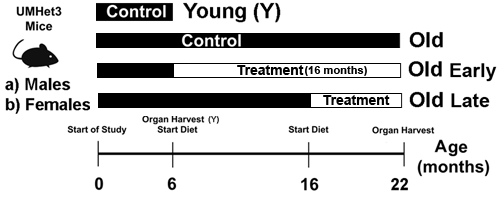

Supplement: Supplementary file 1 — Fig S1 [file ACEL-20-e13345-s001.tif]

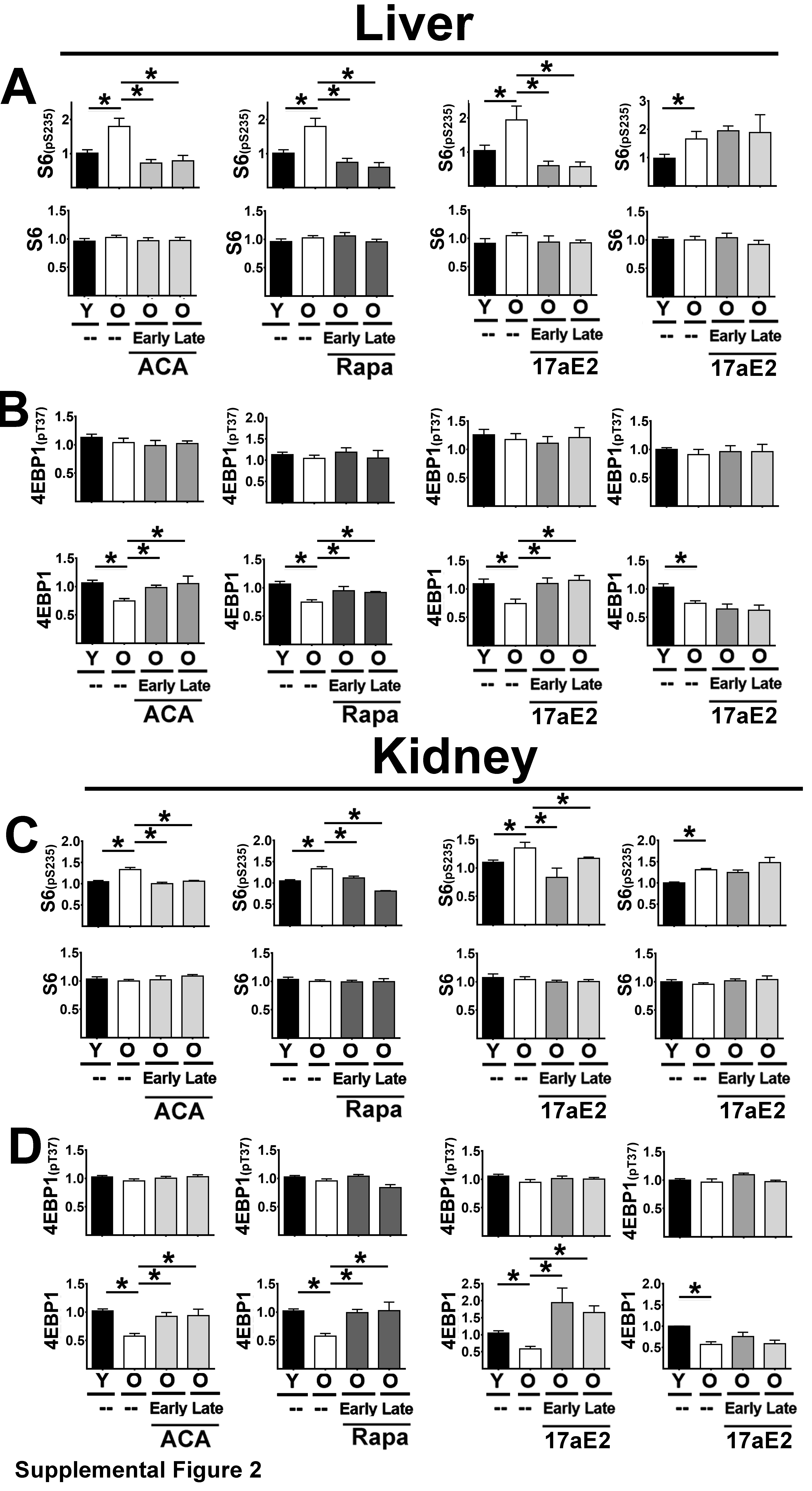

Supplement: Supplementary file 2 — Fig S2 [file ACEL-20-e13345-s003.tif]

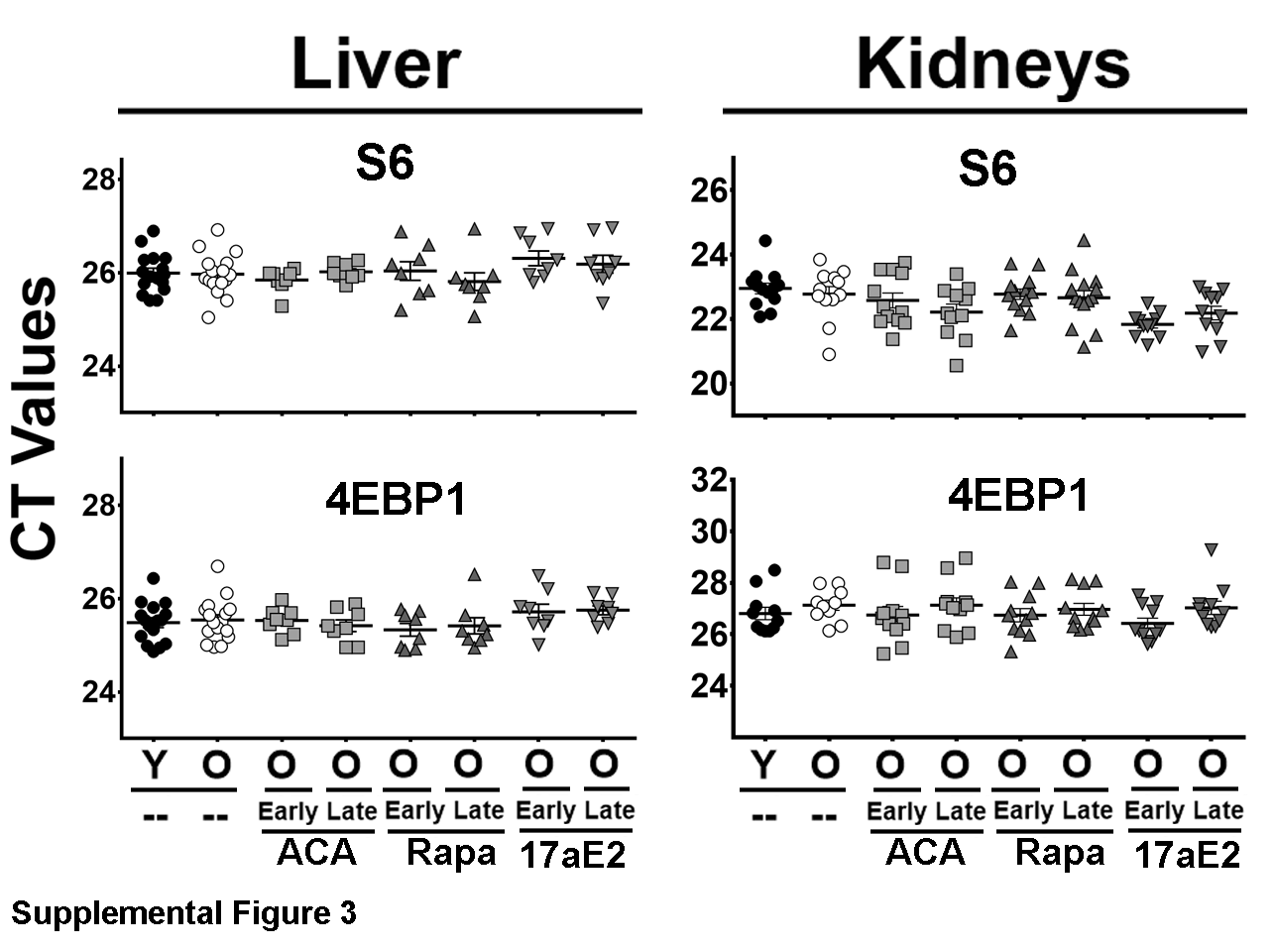

Supplement: Supplementary file 3 — Fig S3 [file ACEL-20-e13345-s002.tif]

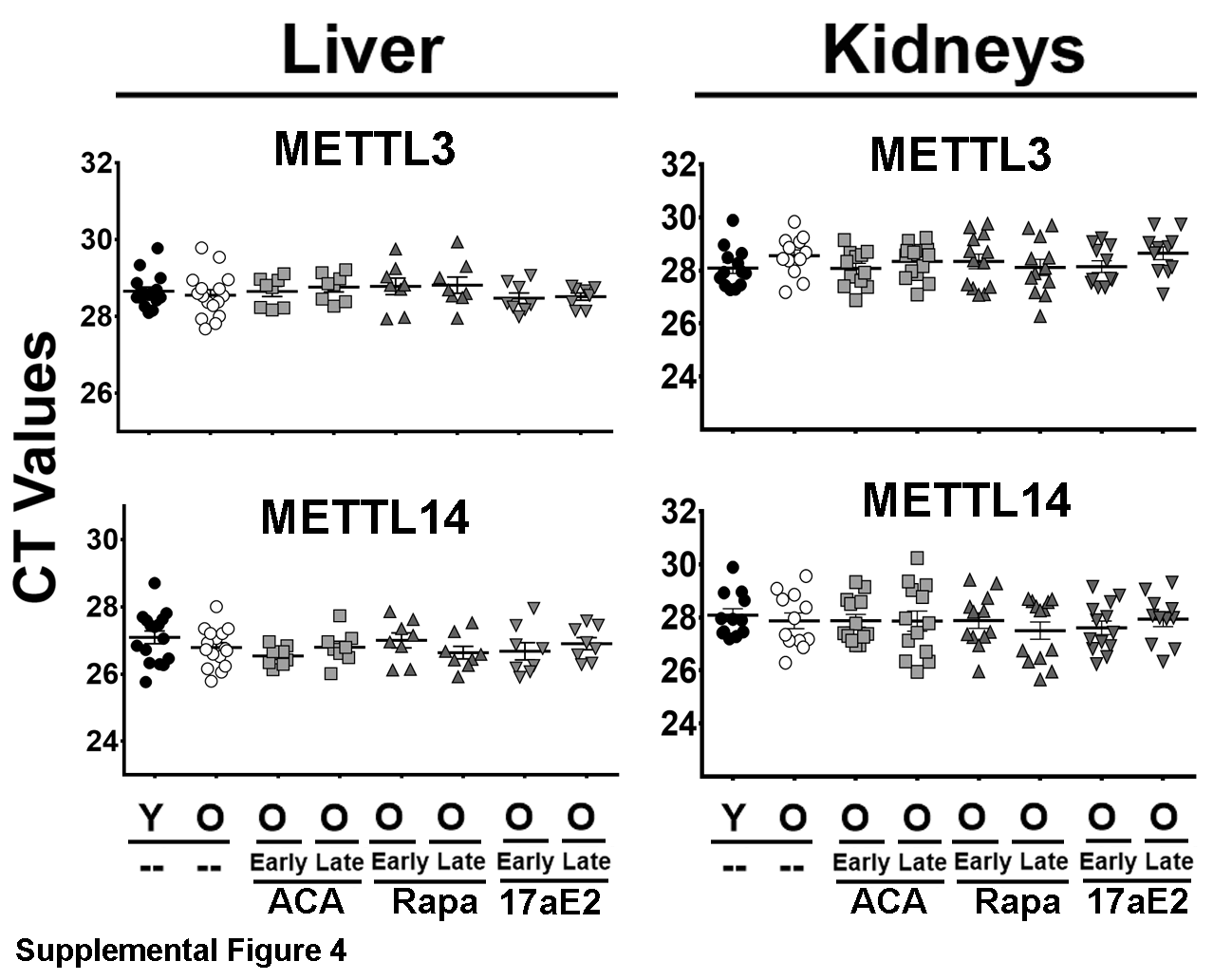

Supplement: Supplementary file 4 — Fig S4 [file ACEL-20-e13345-s004.tif]
