# Supplementary material for: Cap‐independent translation: A shared mechanism for lifespan extension by rapamycin, acarbose, and 17α‐estradiol
Source: Aging Cell. 2021 Mar 20;20(5):e13345. doi: 10.1111/acel.13345 (PMC8135077; doi:10.1111/acel.13345)
Supplement: Supplementary file 5 — Table S1‐S5 [file ACEL-20-e13345-s005.docx]

**Supplemental Table 1. Statistical analysis of age and treatments effects in the CIT target.**

**Supplemental Table 2. Statistical analysis of age and treatments effects in the mTORC1 substrates.**

**Supplemental Table 3. Statistical analysis of age and treatments effects in the METTL3/14 enzymes.**

**Supplemental Table 4. Source of Antibodies**

| **Antibody** | **Source** | **Cat #** |
| --- | --- | --- |
| **pS6(235)** | Cell Signaling | 2211 |
| **S6** | Cell Signaling | 2217 |
| **p4E-BP1(T36/46)** | Cell Signaling | 2855 |
| **4E-BP1** | Cell Signaling | 9644 |
| **pAKT(S473)** | Cell Signaling | 9271 |
| **AKT** | Cell Signaling | 9272 |
| **pNDRG1(T346)** | Cell Signaling | 5482 |
| **NDRG1** | Cell Signaling | 9408 |
| **MGMT** | VisionLab | 3820 |
| **ACTIN** | Santa Cruz | 47778-HRP |
| **TFAM** | Origene | AF26439 |
| **METTL-3** | ThermoFisher | 720347 |
| **METTL-14** | Millipore | ABE1338 |
| **Connexin-20** | ABClonal | 11752 |
| **p38(pT180)** | Cell Signaling | 9211 |
| **p38 MAPK** | Cell Signaling | 8650 |
| **eIF4E(pS209)** | Cell Signaling | 9741 |
| **eIF4E** | Cell Signaling | 9742 |
| **Hsp70** | Cell Signaling | 4872 |

| **Probe** |  | **Mouse Sequence probes** |
| --- | --- | --- |
| **MGMT** | Forward | aaacactgaccccacagagg |
|  | Reverse | aacacagggtgatggagagc |
| **NDRG1** | Forward | cgagagctacatgacgtgga |
|  | Reverse | aagagggggttgtagcaggt |
| **TFAM** | Forward | agccaggtccagctcactaa |
|  | Reverse | aaacccaagaaagcatgtgg |
| **METLL3** | Forward | aaggagccggctaagaagtc |
|  | Reverse | tcactggctttcatgcactc |
| **METLL14** | Forward | tgagagtgcggatagcattg |
|  | Reverse | tctcttcctcctgctgcatt |
| **S6** | Forward | gcaaactctacctcatcctgg |
|  | Reverse | gctagtgtgatctctgccag |
| **4EBP1** | Forward | cggaagataagcgggcag |
|  | Reverse | cagtgtctgcctggtatgag |
| **AKT** | Forward | gccctcaagtactcattccag |
|  | Reverse | acacaatctccgcaccatag |
| **eIF4E** | Forward | ttacagtccttaccacagcac |
|  | Reverse | gttccacagtcgccatcttag |
| **p38MAPK** | Forward | gtgattggtctgttggatgtg |
|  | Reverse | tgagaaactgaacgtggtcg |
| **Hsp70** | Forward | tggtgctgacgaagatgaag |
|  | Reverse | aggtcgaagatgagcacgtt |
| **ACTIN** | Forward | ctaaggccaaccgtgaaaag |
|  | Reverse | accagaggcatacagggaca |

**Supplemental Table 5. qRT-PCR**
